# Acute *Toxoplasma Gondii* Infection in Cats Induced Tissue-Specific Transcriptional Response Dominated by Immune Signatures

**DOI:** 10.3389/fimmu.2018.02403

**Published:** 2018-10-19

**Authors:** Wei Cong, Tania Dottorini, Faraz Khan, Richard D. Emes, Fu-Kai Zhang, Chun-Xue Zhou, Jun-Jun He, Xiao-Xuan Zhang, Hany M. Elsheikha, Xing-Quan Zhu

**Affiliations:** ^1^State Key Laboratory of Veterinary Etiological Biology, Key Laboratory of Veterinary Parasitology of Gansu Province, Lanzhou Veterinary Research Institute, Chinese Academy of Agricultural Sciences, Lanzhou, China; ^2^Department of Marine Engineering, Marine College, Shandong University, Weihai, China; ^3^Faculty of Medicine and Health Sciences, School of Veterinary Medicine and Science, University of Nottingham, Loughborough, United Kingdom; ^4^Advanced Data Analysis Centre, University of Nottingham, Loughborough, United Kingdom

**Keywords:** *Toxoplasma gondii*, host-parasite interaction, transcriptome, differential gene expression, biomarkers

## Abstract

RNA-sequencing was used to detect transcriptional changes in six tissues of cats, seven days after *T. gondii* infection. A total of 737 genes were differentially expressed (DEGs), of which 410 were up-regulated and 327 were down-regulated. The liver exhibited 151 DEGs, lung (149 DEGs), small intestine (130 DEGs), heart (123 DEGs), brain (104 DEGs), and spleen (80 DEGs)-suggesting tissue-specific transcriptional patterns. Gene ontology and KEGG analyses identified DEGs enriched in immune pathways, such as cytokine-cytokine receptor interaction, Jak-STAT signaling pathway, NOD-like receptor signaling pathway, NF-kappa B signaling pathway, MAPK signaling pathway, T cell receptor signaling pathway, and the cytosolic DNA sensing pathway. C-X-C motif chemokine 10 (CXCL10) was involved in most of the immune-related pathways. PI3K/Akt expression was down-regulated in all tissues, except the spleen. The genes for phosphatase, indoleamine 2,3-dioxygenase, Hes Family BHLH Transcription Factor 1, and guanylate-binding protein 5, playing various roles in immune defense, were co-expressed across various feline tissues. Multivariate K-means clustering analysis produced seven gene clusters featuring similar gene expression patterns specific to individual tissues, with lung tissue cluster having the largest number of DEGs. These findings suggest the presence of a broad immune defense mechanism across various tissues in cats against acute *T. gondii* infection.

**Graphical Abstract:**
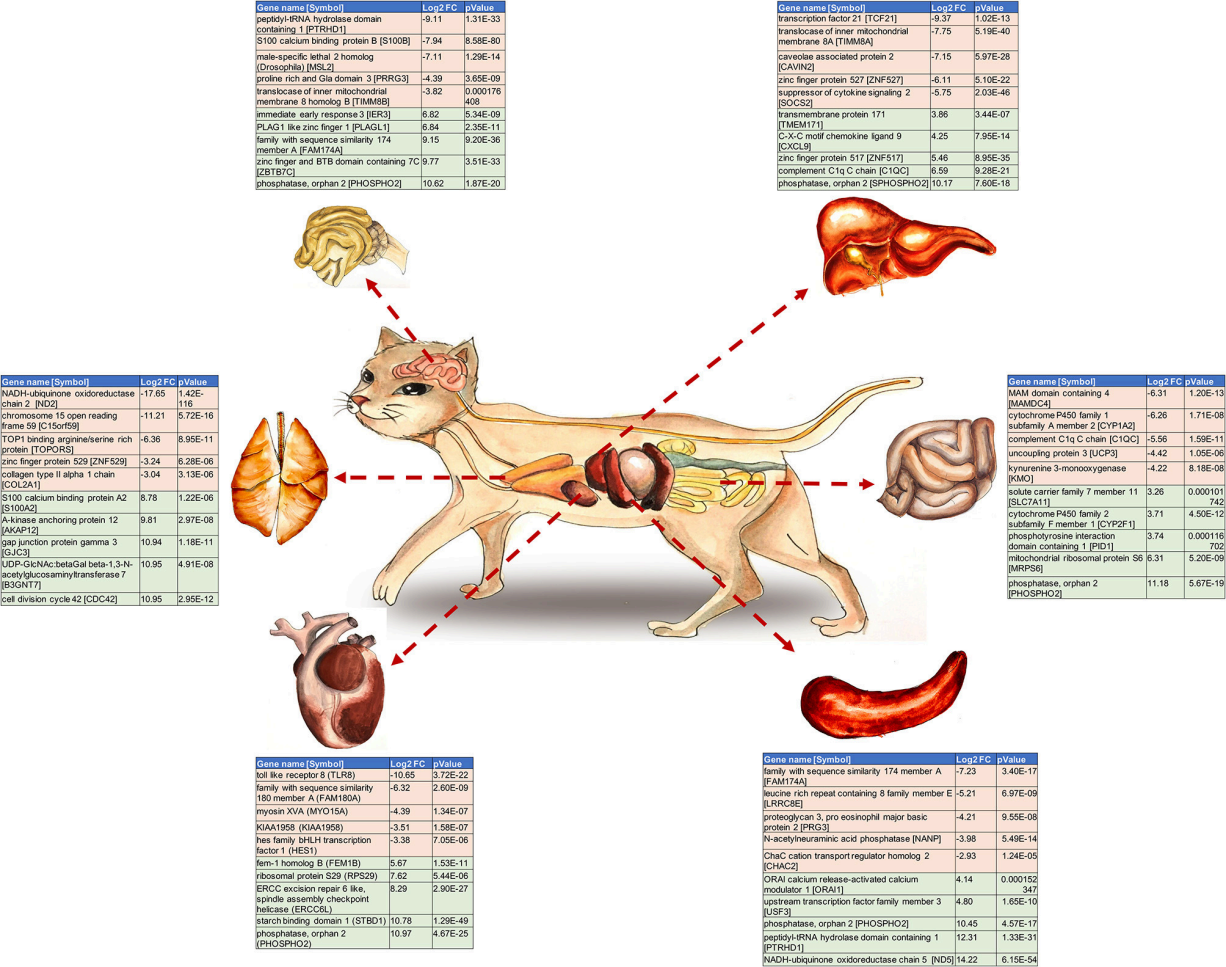
The top 10 Differentially Expressed Genes (DEGs) per examined cat tissue. The gene name and symbol, the log2 fold change and *P*-value for each gene are shown.

## Introduction

The intracellular protozoan *Toxoplasma gondii* infects almost all warm-blooded animals and approximately one-third of the world's human population, causing toxoplasmosis, a serious illness with fatal consequences in immune-compromised individuals and the unborn fetus (1–3). The emergence of drug-resistant parasite strains (4) together with the adverse effects of the currently-available drug therapies (5), and the inability to clear chronic infection highlight the need for improved treatment strategies to combat toxoplasmosis in humans and animals. *T. gondii* has a complex lifecycle, wherein the parasite undergoes asexual reproduction in the intermediate host and sexual reproduction in the definitive host (members of the Felidae family). Cats acquire infection by ingesting prey tissue containing parasite cysts or, more rarely, oocysts. Prenatal infection may also occur in cats and humans (2, 6). Infected cats discharge oocysts (the product of parasite sexual reproduction in the cat's intestine) in their feces. The ability to accommodate both sexual and asexual reproduction of *T. gondii*, makes cats a significant source of infection to humans and animals (1, 7). *T. gondii* is a food-borne pathogen acquired via oral infection; however, it has a dispersive nature and can disseminate throughout the cat's body to infect multiple tissues (8–14).

Recognizing early transcriptional signatures of infection, while knowing the factors that determine tissue susceptibility to *T. gondii* infection in cats, would assist in planning preventative measures against environmental contamination with oocysts. Previous investigations have provided insights into the transcriptome of many intermediate host species during *T. gondii* infection (15–21). However, knowledge of the mechanisms that underpin the feline host transcriptional response to *T. gondii* remains poorly understood; and no genome-wide expression profiling of multiple tissues from cat, has been reported. Single-tissue gene expression can provide information on how a specific tissue responds to infection; however, understanding the patterns of gene expression across various tissues may advance our understanding of *T. gondii* molecular-pathogenesis events occurring during acute infection in cats and reveal the mechanisms by which the definitive host counters a complex infection such as *T. gondii*. RNA sequencing (RNA-Seq) of the whole transcriptome has proved a powerful and versatile tool for global gene expression analysis (22–24), enabling a comparison of transcriptomes between the merozoite and tachyzoite stages of *T. gondii* infecting the cat's intestine (25).

Here, we hypothesized that tissues of *T. gondii*-infected cats exhibit characteristic transcriptional signatures which are dominated by a number of genes, and may be exclusive to a particular tissue with variance across tissues from the same individual. Differential gene expression and gene clustering analyses were performed on six tissues, individually or combined, in cats infected with *T. gondii*. Our studies provided novel information about the transcriptomic landscape of the major tissue types in cats during acute *T. gondii* infection and revealed tissue responsive signatures during acute *T. gondii* infection.

## Materials and methods

### Ethics approval

All efforts were made to minimize suffering and to reduce the number of animals in the experiment. All work with live *T. gondii* was performed at biosafety level 2 and the animal experimental protocols were approved by The Animal Administration and Ethics Committee of Lanzhou Veterinary Research Institute, Chinese Academy of Agricultural Sciences (Protocol Permit Number: LVRIAEC-2014-001).

### Animal husbandry

Twelve domestic cats (*Felis catus;* 2 to 3-months-old; Chinese Li Hua breed) were purchased from a local breeder and housed individually in a controlled environment. Six cats each from two litters– were randomly allocated into four groups (2x infected and 2x control) with three cats per group. All of the cats were tested negative for feline immunodeficiency virus and feline leukemia virus using SNAP FIV/FeLV Combo Test (IDEXX, Westbrook, US), and feline calicivirus and feline parvovirus by ELISA KIT (NuoYuan, Shanghai, China) prior to the experiment. Also, cats tested seronegative for the presence of specific anti-*T. gondii* antibodies using indirect fluorescent antibody test (IFAT). Cats were supplied with a commercial diet (Royal Canine Inc., St. Charles, MO, USA) and water ad libitum during the 3 weeks prior to experimentation. During the experiment cats were fed a maintenance diet, based on their daily energy requirement.

### Parasite strain, infection and sample collection

*T. gondii* Pru strain (genotype II) was maintained by passage through Kunming mice (26). The number of *T. gondii* cysts (determined using an optical microscope) was adjusted to 100 cysts per 1ml phosphate buffered saline (PBS, pH 7.4). Each experimental animal was infected with 100 cysts in 1 ml PBS by intragrastric inoculation. The enteroepithelial sexual cycle of *T. gondii* is completed within 3-10 days post ingestion of *T. gondii* cysts. This period can be extended to >18 days if cats are infected by oocysts. Control cats were sham-infected with 1 ml PBS only. Six different tissues (brain, heart, liver, lung, small intestine, and spleen) were collected from cats seven days after infection, in order to allow sufficient time for infection to establish. Tissue collection was performed as a terminal procedure from cats under deep isoflurane anesthesia, and unresponsiveness to all stimuli. Collected tissue samples were rinsed in saline, flash frozen in liquid nitrogen and stored at −80°C until processing.

### Confirmation of infection

Total genomic DNA was extracted from all harvested tissues using TIANamp Genomic DNA kit (TianGen™, Beijing, China). A PCR assay targeting *T. gondii* B1 gene was used to detect *T. gondii* infection in all cat tissues (27, 28). The PCR products were subjected to electrophoresis on ethidium bromide-stained 2% agarose–Tris-acetate-EDTA gels, and the banding pattern was visualized by UV transillumination. All of the electrophoresed PCR products were run with positive and negative control. *T. gondii* genotype was confirmed by PCR-restriction fragment length polymorphism (RFLP) analysis of the positive amplicons (29).

### RNA extraction and qualification

Total RNA was extracted individually from the six tissues of the cats using TRIzol Reagent according to the manufacturer's protocol (Invitrogen, CA, USA). The RNA was checked for degradation and contamination using 1% agarose gels. RNA purity was evaluated with a NanoPhotometer® spectrophotometer (IMPLEN, CA, USA) and RNA concentration was measured using the Qubit® RNA Assay Kit in Qubit® 2.0 Flurometer (Life Technologies, CA, USA). RNA integrity was assessed using the RNA Nano 6000 Assay Kit of the Agilent Bioanalyzer 2100 system (Agilent Technologies, CA, USA).

### cDNA library preparation and illumina deep sequencing

RNA samples that conformed with Quality Control checks (QC) were used in the transcriptome-sequencing (RNA-seq) analysis. Samples were run in duplicate, and each RNA template consisted of three pooled biological replicates from the same group. 3 μg RNA per sample was used as input material for RNA-seq library preparation using NEBNext® Ultra™RNA Library Prep Kit for Illumina® (NEB, USA). Index codes were added to correlate sequences to their respective samples. The mRNA was purified from total RNA using poly-T oligo-attached magnetic beads. Fragmentation was carried out using divalent cations under elevated temperature in NEBNext First Strand Synthesis Reaction Buffer (5X). First strand cDNA was synthesized using a random hexamer primer and M-MuLV Reverse Transcriptase (RNase H-). Second strand cDNA was synthesized using DNA Polymerase I and RNase H. Remaining overhangs were converted into blunt ends using exonuclease/polymerase. Following adenylation of the 3′ends of DNA fragments, NEBNext Adaptor with hairpin loop structures were ligated to prepare for hybridization. In order to select cDNA fragments of 150~200 bp in length, library fragments were purified using the AMPure XP system (Beckman Coulter, Beverly, USA). Then, 3 μl USER Enzyme (NEB, USA) were used with size-selected, adaptor-ligated cDNA at 37°C for 15 min followed by 5 min at 95°C before PCR, which was performed with Phusion High-Fidelity DNA polymerase, Universal PCR primers and Index (X) Primer. PCR products were purified (AMPure XP system) and the library quality was assessed with the Agilent Bioanalyzer 2100 system. The clustering of index-coded samples was performed on a cBot Cluster Generation System using TruSeq PE Cluster Kit v3-cBot-HS (Illumia) according to the manufacturer's instructions. Following cluster generation, library preparations were sequenced on an Illumina Hiseq 2500 platform and 125 bp paired-end reads were generated.

### Quality trimming of illumina paired-end reads

Before read alignment, all the data files (Fastq) were adaptor trimmed using “scythe” (v0.994 BETA) (https://github.com/vsbuffalo/scythe) and quality trimmed using the library “sickle” (v1.33) (https://github.com/najoshi/sickle).

### Read alignment and transcript assembly

Index of the reference genome was built using Hisat2 (v2.1.0) (30). Trimmed paired-end reads were aligned to the reference genome using Hisat2 for expression estimation. StringTie (v1.3.3) was then used to assemble the read alignments into known transcripts for each sample (31). In addition, StringTie also produces a gene abundance table (FPKM and TPM), which was used for clustering analysis.

### Tissue-specific differential expression analysis

A combined read count table at the gene level for all the samples was generated using a python script available from StringTie. The Bioconductor package edgeR (v3.18.1) (32) was used to identify the differentially expressed (DE) genes per-tissue condition (infected vs. uninfected; two biological replicates per condition). Genes with a 5% false discovery rate (FDR < 0.05) and log fold change (logFC) ≥ 1 were considered differentially expressed.

### Gene ontology (GO) and pathway analyses

The Bioconductor package GOstats (v2.42.0) (33) was used to test for over-representation of GO terms using a hypergeometric test (hyperGTest). Orthologues for cat gene-sets were found using Ensembl BioMart against human data; then the cat gene orthologues were used for gene ontology analysis. GO terms with a corrected *P* < 0.05 were considered significantly enriched. Pathway analysis was performed using bioconductor package “pathview” (v1.16.5), which implements Kyoto Encyclopedia of Genes and Genomes (KEGG) pathways. The significance level of enrichment of KEGG pathways was identified using FDR < 0.05 and a corrected *P* < 0.05.

### K-means clustering

The 21,890 genes identified through HiSat2 and StringTie were subjected to clustering analysis. In detail: the FPKM values of the genes from both replicates of each tissue and each treatment (e.g., rep 1 and rep 2 of infected brain tissues) were averaged; the log of the ratio between infected and non-infected conditions for each tissue was calculated; then, two subsequent k-means clustering analyses were performed [MeV_4_8 v10.2 (Multi Experiment viewer)] using Euclidean distance and k = 10 number of clusters. A stringent expression cutoff was applied to each sub-group of genes to discard background noise. Specifically, in each of the six generated clusters where genes showed increased expression levels concentrated on a single infected tissue, only those genes with FPKM values >1 in the specific infected condition were considered as expressed and selected for further analysis. To visualize gene expression in each cluster, bean plots (which represent the actual distribution of the individual data sets) were produced using BoxPlotR: a web-tool for generation of box plots.

### Quantitative real time (RT)-PCR

Total RNA was extracted from various cat tissues using the TRIzol method (Invitrogen) and reverse-transcripted to single strand cDNA using the GoScript^TM^ Reverse Transcription System (Promega, MI, USA). GoTaq® qPCR Master Mix (Promega, MI, USA) was used to perform RT-PCR reactions in a QIAGEN's real-time PCR cycler (Rotor-Gene Q). Amplification reactions were performed under the following conditions: 2 min at 95°C, 40 cycles of 95°C for 15s, 55°C for 30s, and 72°C for 30s. All quantitative measurements were carried out in triplicate and normalized to the housekeeping gene glyceraldehyde-3-phosphatedehydrogenase (*GAPDH*) for every reaction (34). Twelve significant DEGs were selected to validate the sequencing data. Primers used for RT-PCR are listed in Table 1. The mRNA fold change was calculated using the following equations (35):

(1)     CΔT=CΔT(target)−CΔT(GAPDH);CΔΔT=CΔT(infected)−CΔT(control);mRNA fold change=2−ΔΔCT

**Table 1:** Gene names and primers used in qRT-PCR analysis.

**Gene**	Primer name^*^	**Primer sequence (5′to 3′)**
ADAM11	ADAM11-F	5′-CTGTGGCTTCCTCCTCTGTGT−3′
	ADAM11-R	5′-TTGCCCTGGTGGTAGAAGGT−3′
APOA2	APOA2-F	5′-CGGTGACTGACTACGGCAAG-3′
	APOA2-R	5′-TAACTGCTCCTGGGTCTTCTCAA−3′
MEP1A	MEP1A-F	5′-CACCATCATCAACATCCTGTCTC−3′
	MEP1A-R	5′-AAGGAAGGTCTGAAGTAGCAAAGGT-3′
ENO4	ENO4-F	5′-TGCATCTCTGTGTTGGTTATGCT−3′
	ENO4-R	5′- CGAAGGGCTACATACCGATTTTAC-3′
IGFI	IGFI-F	5′-GAGAGGAGTGGAAAACGCAGA−3′
	IGFI-R	5′-AGCGGTGAGTCCAAGACAGAG−3′
GKN2	GKN2-F	5′-CATGCTCCTCTACCACGGTTT−3′
	GKN2-R	5′-GCAGGGATGGCTTTATGTTTC−3′
GBP5	GBP5-F	5′-GCTAAAGGAAGGCACCGATAAA−3′
	GBP5-R	5′-AGTGAGCAGGAGAGTCGAAGATAAA−3′
OAS1	OAS1-F	5′-AGCCATCCACATCATCTCCAC−3′
	OAS1-R	5′- AGAGCCACCCTTGACCACTTT-3′
IDO1	IDO1-F	5′-GAACCAAGGCGGTGAAGATG−3′
	IDO1-R	5′-GCATAAACCAGAATAGGAGGCAGA−3′
PI16	PI16-F	5′-CTGCCAGAACTGTCTGCCTCT−3′
	PI16-R	5′-GTCCTTCATCTGCCCCTCAC−3′
ACTG2	ACTG2-F	5′-AACAGGGAGAAGATGACCCAGA−3′
	ACTG2-R	5′-CCAGAAGCATAGAGAGAGAGCACA−3′
ANKFN1	ANKFN1-F	5′-ATACCTCTACACCAGGCAAGGAAC−3′
	ANKFN1-R	5′-GCAGGGAGCAGGAGAAGAAA−3′
GAPDH	GAPDH(CAT)-F	5′-AAGCCCATCACCATCTTCCA−3′
	GAPDH(CAT)-R	5′-TTCACGCCCATCACAAACA−3′

* Forward (F) and reverse (R) primers.

*ADAM11, ADAM metallopeptidase domain 11; APOA2, apolipoprotein A2; MEP1A, Meprin A Subunit Alpha; ENO4, enolase family member 4; IGFI, Insulin-like growth factor 1 level; GKN2, gastrokine 2; GBP5, Guanylate Binding Protein 5; OAS1, 2'-5'-Oligoadenylate Synthetase 1; IDO1, indoleamine 2,3-dioxygenase 1; PI16, Peptidase Inhibitor 16; ACTG2, actin, gamma 2; ANKFN1, ankyrin repeat and fibronectin type III domain containing 1; GAPDH, glyceraldehyde-3-phosphate dehydrogenase*.

## Results

### Presence of *T. gondii* infection in cats

Parasite dissemination from the intestine to other tissues, both close to the inoculation site (small intestine, liver, and spleen) and to distantly placed organs (brain, heart, and lungs) was confirmed seven days post infection. *T. gondii B1* gene-based PCR analysis detected the parasite DNA in the brain, heart, liver, lung, small intestine, and spleen of all infected cats (Figure S1). The parasite load did not seem to vary across the cat tissues. However, it is possible that the parasite load may vary over the course of infection. *T. gondii* DNA was not detected in any tissue of the control cats. All positive amplicons that were characterized by RFLP produced a restriction fragment pattern that correlated with *T. gondii* genotype II.

### General features of the transcriptome data

Transcriptome-sequencing analysis generated ~143 million sequence reads (125 bp in length) from 24 libraries. After quality control analysis and the removal of low quality reads, ~170 Gb clean reads were obtained, with an average of 7 Gb clean reads/tissue. Less than 95% of the clean reads had Phred-like quality scores at the Q20 level and GC content of about 50% (Table S1). The clean reads were mapped to the genome of *Felis catus*. The majority of the clean reads were distributed in the exon region, with fewer in the intergenic region and the intron region. Approximately 80% of the clean reads were unique (Table S2); and subsequent analyses were based on these uniquely mapped reads.

### Infection induced significant alterations in gene expression

**Figure 1:**
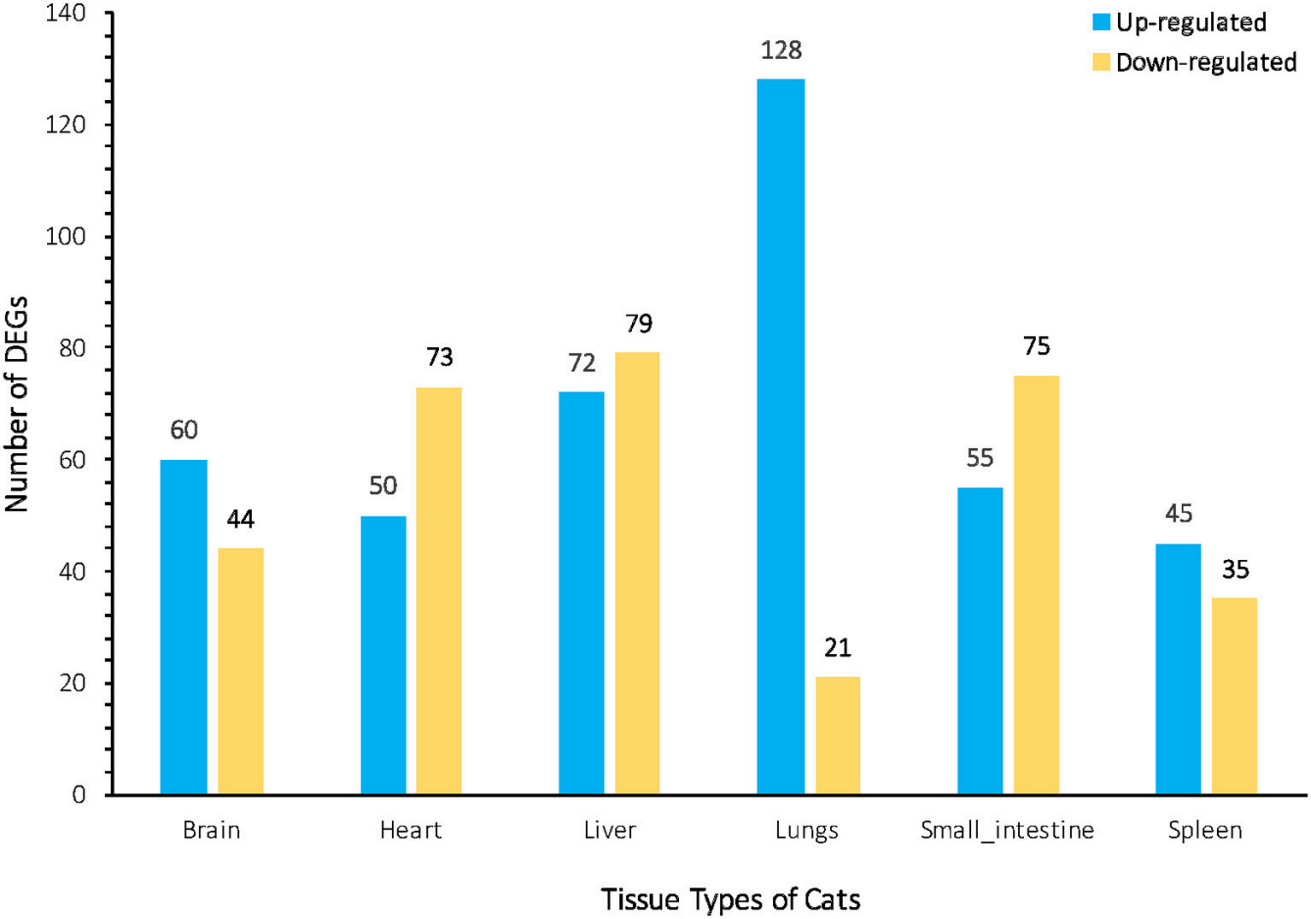
Bar plot representation of the differentially Expressed Genes (DEGs) across the cat tissues after acute *T. gondii* infection. The numbers of up-regulated and down-regulated DEGs assigned to each cat tissue are indicated above the bars. The greatest changes in DEGs between infected and uninfected tissues were observed in the liver and lung.

**Figure 2:**
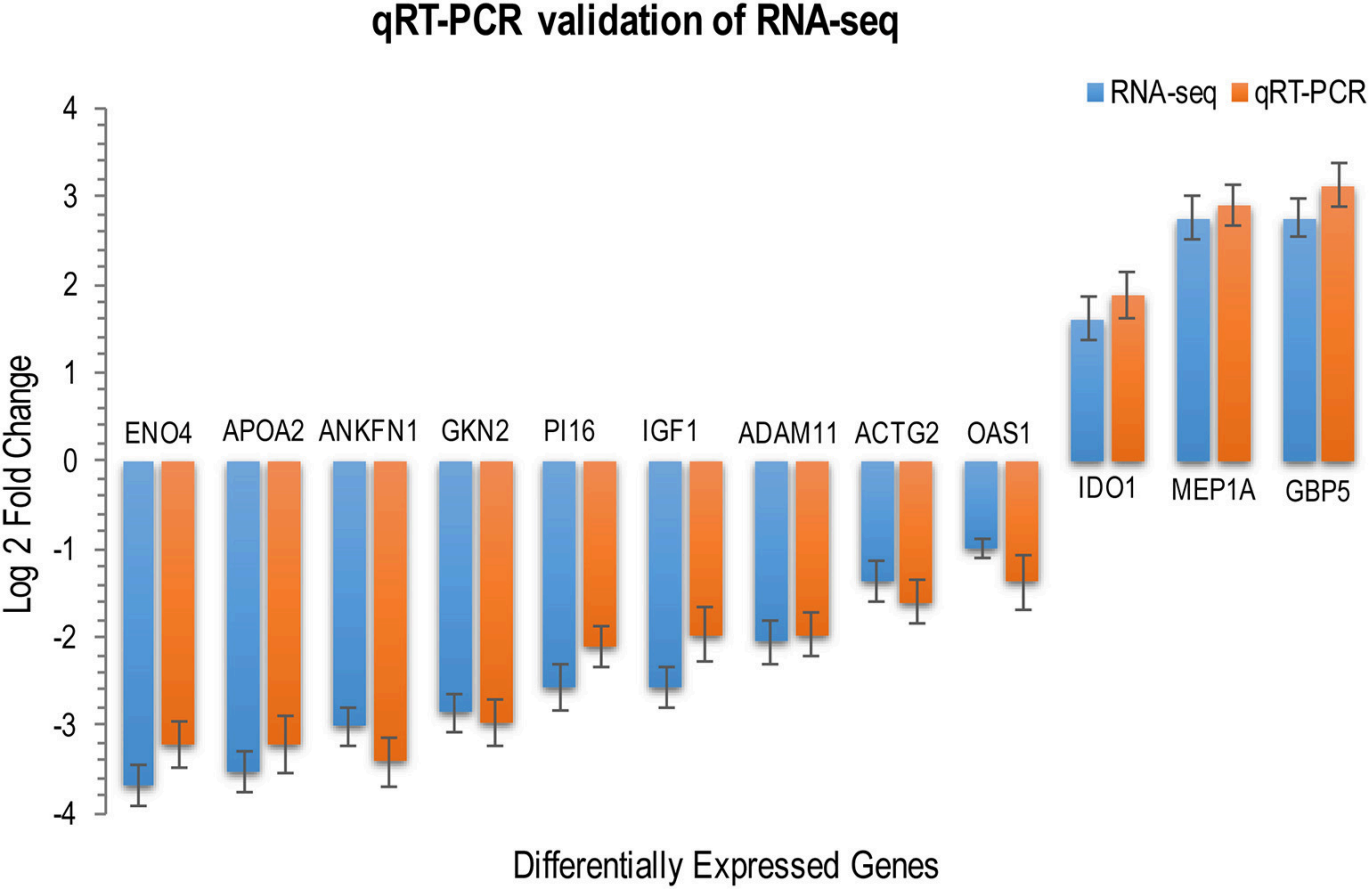
RNA-seq transcriptome analysis and quantitative, real-time RT–PCR produced similar gene expression profiles. The expression levels of 12 DEGs across various cat tissues were determined by qRT-PCR for validation of RNA-seq data. Relative expression levels were calculated using the the ΔΔC*T* threshold cycle (C*T*) method and *GAPDH* as the reference gene. RNA-seq data are mean of two biological replicates + standard deviation (SD) of normalized read counts. qRT–PCR data are mean of three biological replicates + SD. *P* values are calculated with unpaired, two-tailed *t*-test. The height of the bars represents the log-transformed median fold changes in gene expression between infected and uninfected cats.

We investigated the distribution of gene expression values across the six tissues by fragment Per Kilobase of exon per Million mapped reads (FPKM) (Table S3). ~47% of the expressed genes had low expression values (0 < FPKM ≤ 1). The number of genes with moderate expression values (1 < FPKM ≤ 60) and high expression values (FPKM > 60) accounted for ~53% of the total annotated genes. Genes with FPKM ≥1 [ranging from 12,195 (43.80%) to 16,734 (60.10%)] in the six tissues were considered expressed genes. We used the Bioconductor package edgeR (v3.18.1) to identify DEGs in each body tissue of the infected and uninfected cats. A total of 737 genes were differentially expressed in infected vs. uninfected cats, of which 410 were up-regulated and 327 were down-regulated. Large differences in gene expression were observed between cat tissues, indicating heterogenities in the response of cat tissues to *T. gondii* infection. Liver exhibited the highest number of DEGs (151 genes) compared to lung (149 genes), small intestine (130 genes), heart (123 genes), brain (104 genes), and spleen (80 genes) (Figure 1). DEGs of each tissue are listed in Table S4. We also used quantitative real-time PCR to validate the expression levels of representative genes, across cat tissues, detected by RNA-sequencing analysis (Figure 2).

### Gene ontology (GO) enrichment and functional annotation analyses

GO enrichment analysis was used to identify the significantly enriched GO terms in all DEGs using the GOseq R package. The enriched GO terms of each tissue are shown in Table S5. All DEGs were mapped to terms in the KEGG database. DEGs and KEGG pathways related to immune response were highly represented and are summarized in Table S6.

### Signatures of gene co-expression

**Figure 3:**
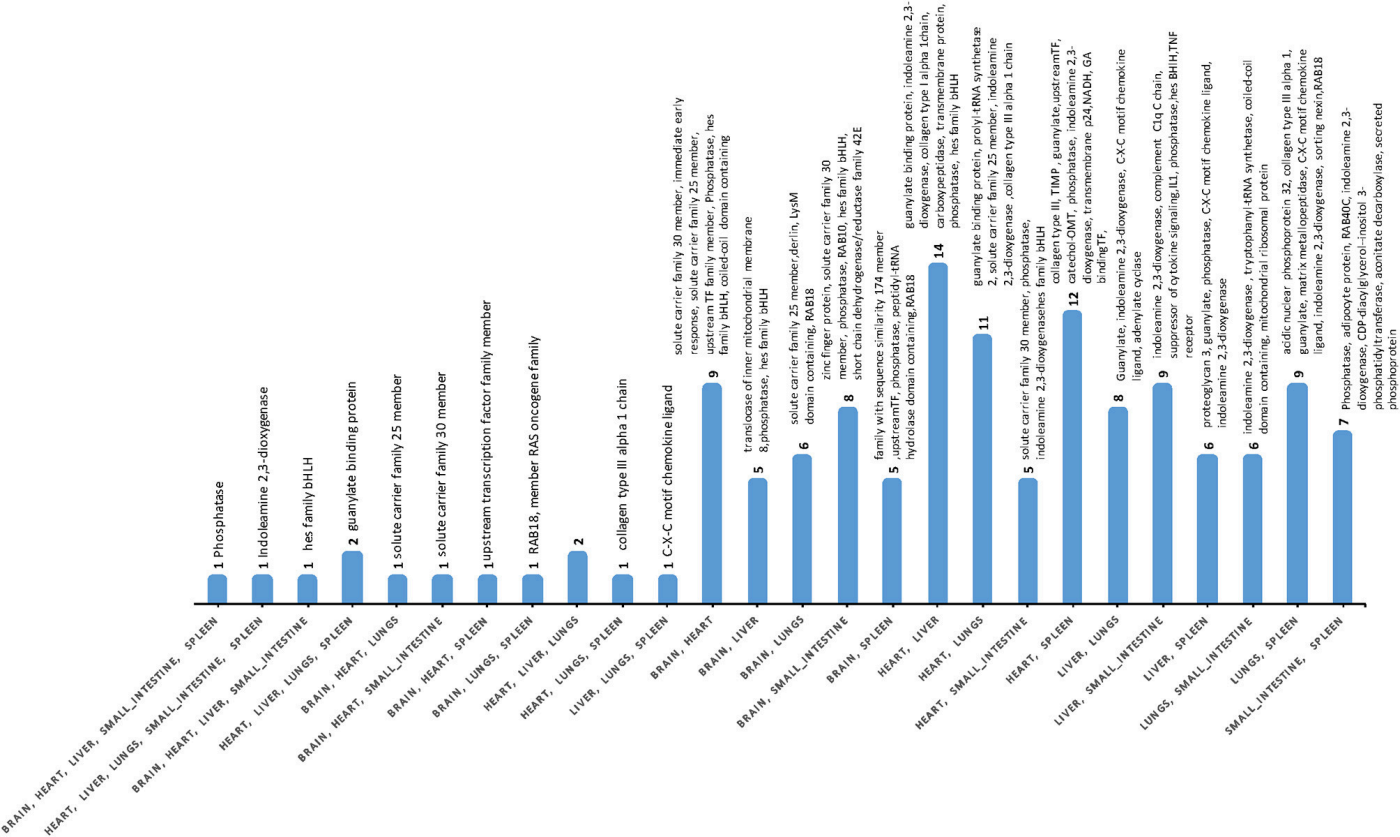
The number of co-expressed DEGs across cat tissues. Phosphatase and indoleamine 2,3-dioxygenase were co-expressed in five tissues, whereas Hes Family BHLH Transcription Factor 1 and Guanylate-binding protein 5 were co-expressed in four tissues. Only fully annotated genes are shown.

Differences were detected between the transcriptomes of cat tissues, with significant variations in gene expression between infected and uninfected tissues (Table S4). Gene co-expression analysis of DEGs indicated that phosphatase and indoleamine 2,3-dioxygenase (IDO) were co-expressed in five tissues (brain, heart, liver, small intestine, and spleen); while the HES1 (Hes Family BHLH Transcription Factor 1) was co-expressed in brain, heart, liver, and small intestine. Guanylate-binding protein 5 was detected in heart, liver, lung, and spleen (Figure 3, Table S7). Expression patterns and pathways associated with co-expressed DEGs across the various cat tissues are shown (Figure S2).

### Global gene regulation during *T. gondii* infection

**Figure 4:**
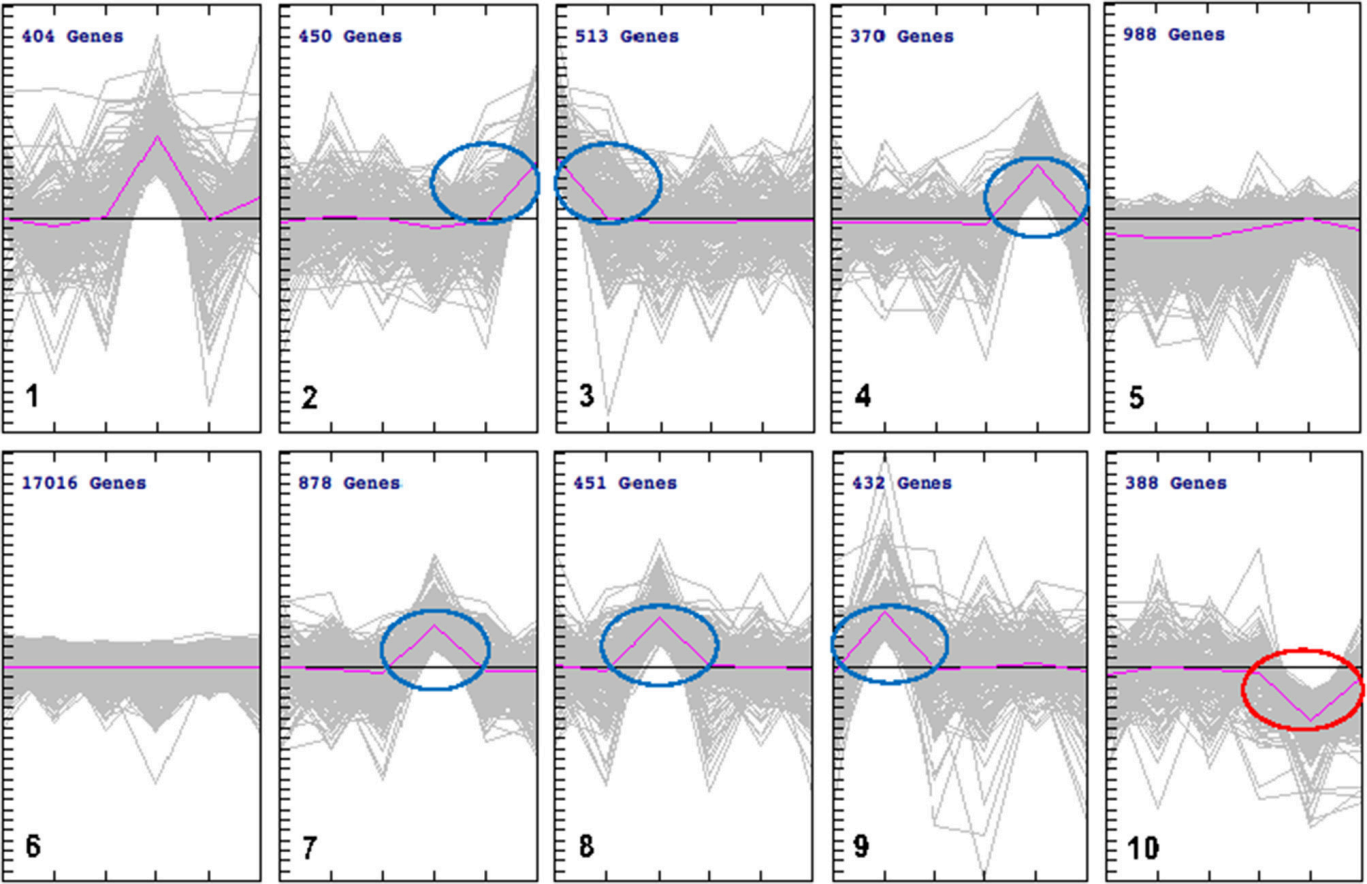
k-Means clustering patterns of the differentially expressed genes across cat tissues. All DEGs were clustered into 10 groups using k-means clustering method and visualized with TM4 software. The pink line shows average expression z-scores to visualize the dominant expression trend of each cluster. Each line in the figure represents an expression value of the corresponding gene. The horizontal axis indicates the type of cat tissues and the vertical axis is the log2 expression ratio. Fold expression changes between various tissues from infected and uninfected cats were calculated using the log2 ratios. The numbers of genes for each cluster are indicated. The clusters included DEGs whose expression was either up regulated (clusters 2, 3, 4, 7, 8, and 9) or down regulated (cluster 10). There were no changes in gene expression observed in clusters 5 and 6. DEGs in cluster 1 was not considered due to lack of specificity to a single tissue.

**Figure 5:**
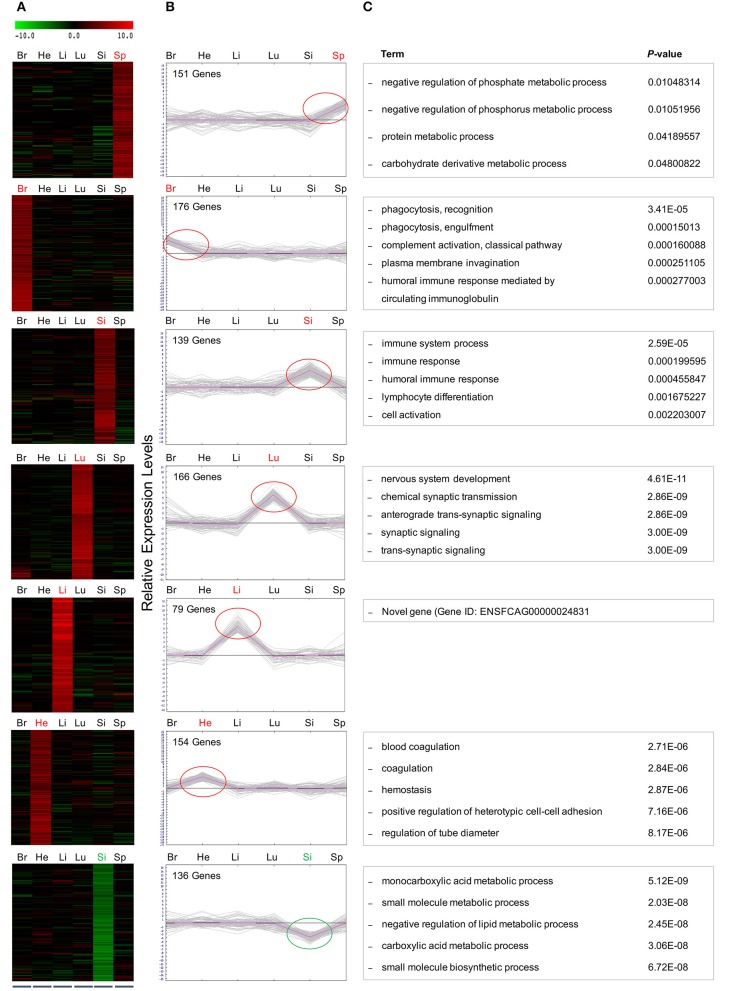
Hierarchical and K-mean clustering analysis of the DEGs within clusters. Differentially expressed, co-regulated genes in each cluster were grouped using k-means clustering. Average cluster size varied considerably among tissues with lung containing the largest cluster with 75 genes. The smallest cluster was found in the liver, averaging 1 gene per cluster. The DEGs clustered into 7 major groups, demonstrated in **(A)** heat map and **(B)** graphical format, based on patterns of gene expression across the differing cat tissues. Red and green circles indicate the tissue-specific up- and down regulated genes, respectively. Negative values indicate decreased expression, and positive values indicate increased expression. **(C)** GO analysis of DEGs within clusters after FPKM filtering identified the top associated enriched GO terms with corresponding enrichment *P-*values, shown on right.

We employed cluster analysis, which was based on the partition of genes into clusters according to the log-ratios of gene expression between infected and uninfected tissues using a two-tier K-means algorithm. This analysis identified a set of 10 gene groups each characterized by a unique pattern. The patterns corresponding to individual clusters are visualized as line plots (Figure 4). Seven of these 10 clusters showed significant expression levels: spleen (cluster 2); brain (cluster 3); lung (cluster 7); liver (cluster 8), and heart (cluster 9), included up-regulated genes. Small intestine tissue produced two clusters with opposite regulation: cluster 4 (up-regulated genes) and cluster 10 (down-regulated genes). Two clusters (5 and 6) did not exhibit gene expression patterns specific to any tissue. To isolate the most representative genes in each cluster, a second clustering analysis was performed, focusing on genes contained in each of the seven clusters identified by the initial cluster analysis. Ultimately, seven groups of tissue-specific genes were identified, and are shown in heat maps and graphical formats, based on patterns of expression of individual genes across various tissues (Figures 5A,B).

### Refined gene clusters after FPKM filtering

Here, a stringent expression cutoff was applied to each sub-group of genes to remove background noise. In each of the six clusters where genes showed increased expression concentrated on a single infected tissue, only those genes with FPKM values >1 in the infected condition were selected for further analysis. Five of these clusters contained up-regulated genes in the spleen (6 genes), brain (13 genes), lung (75 genes), liver (1 gene), and heart (6 genes). However, the small intestine produced one cluster of 19 up-regulated genes and one cluster of 21 down-regulated genes. GO analysis of genes within these seven clusters provided new insights into biological processes, cellular processes and molecular functions regulated in cats during *T. gondii* infection. The five most highly associated biological process terms with the enrichment *P-*values of each cluster are shown (Figure 5C).

KEGG analysis of the DEGs within these seven final clusters was performed to gain more insight into the biological processes influenced during infection. We found no similarity in gene annotation in any cluster in any of the tissues examined. In small intestine cluster, KEGG analysis identified networks associated with hematopoietic cell lineage, metabolism of xenobiotics by cytochrome-P, chemical carcinogenesis and cytokine-cytokine receptor interaction. Lung cluster showed high expression levels of genes involved in nicotine addiction, biosynthesis of unsaturated fatty acids, long-term potentiation, glycosaminoglycan biosynthesis - heparan sulfate/heparin, cell adhesion molecules, insulin secretion, GABAergic synapse, fatty acid metabolism, amyotrophic lateral sclerosis, and adrenergic signaling in cardiomyocytes. The heart cluster showed high expression levels in complement and coagulation cascades and platelet activation. Small intestine cluster showed high expression levels of genes involved in metabolic metabolism, drug metabolism and fatty acid and amino acid metabolism. Genes with different expression patterns across the seven clusters are shown in Table S8. All of the analyzed clusters contained genes annotated to GO terms from the three ontologies at roughly equivalent levels. However, we found considerable bias in some tissues in regards to the representation of clustered genes among the three ontologies (Figure S3). The high proportion of genes annotated to biological process, reflects a trend toward clustering of metabolic and immune pathways. The most striking example was in the lung cluster, where 253 terms were found in the biological process.

### Tissue-specific changes in gene expression

**Figure 6:**
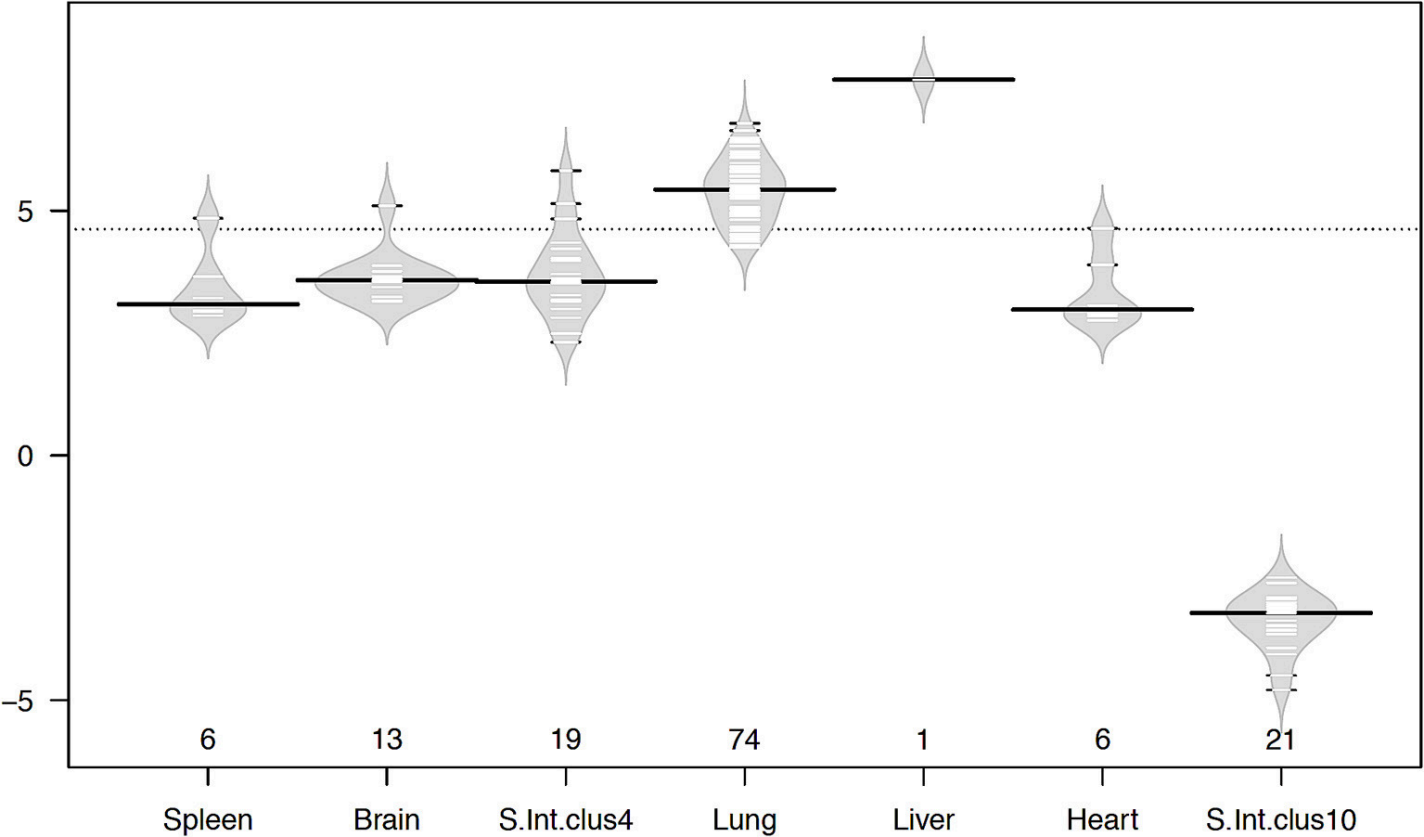
Beanplots showing variation in the magnitude of gene expression within clusters in infected vs. uninfected tissues. The x-axis shows the number of genes of each cluster in the corresponding tissue. The y-axis indicates the average log2 fold change in gene expression. The width of the plot represents the distribution of data, short lines inside the shapes depict individual data points and heavy horizontal lines show the medians within each cluster, while the dotted line indicates the overall average. Plots were drawn using the R beanplot package.

Even though the number of genes in each cluster was small, large-magnitude gene expression changes between infected and uninfected was observed in most of the tissues (Figure 6). The liver gene cluster had the highest magnitude (7.68 fold), followed by lung (5.44 fold), brain (3.58 fold), small intestine cluster 4 (3.55 fold), spleen (3.09 fold), and heart (2.98 fold); whereas small intestine tissue cluster 10 had a magnitude of −3.22 fold. Apart from the liver with only on gene, lung, brain, small intestine, and heart showed large magnitude transcriptional changes occurring in a small number of genes. This suggests that changes induced by *T. gondii* infection seem to rely on a small number of genes, but with large transcriptional changes. The lung was the tissue showing the greatest number of genes with the largest magnitude of transcriptional change. Among all highly expressed genes, the metallothionein 3 (MT3) gene showed the highest expression in the infected lung, and it was also one of the genes with the highest log ratio between infected and uninfected tissues. Metallothionein-3 protein encoded by MT3 gene binds metals both in natural (such as Zn, Cu, Se) and xenobiotic (such as Ag, Cd) conditions, conferring a protective role against metal toxicity and oxidative stress.

## Discussion

The influence of *T. gondii* infection on the transcriptome of different tissue of cats is largely unknown. In this study, we examined the transcriptomes of liver, lung, small intestine, heart, brain, and spleen tissues of cats, seven days post infection. Inter-tissue transcriptome comparison revealed that the levels of DEGs were highest in the liver (151 genes) and lungs (149 genes), indicating a significant gene transcriptional response to infection in the liver and lung compared to other feline tissues. This result supports previous reports, wherein liver and lung seemed to be the most likely tissues to be involved in clinical cases of toxoplasmosis with a rapid fatal outcome (11–13, 34, 36). It is plausible that the timing and duration of infection can influence host tissue transcriptional response to the parasite, as previously indicated by the difference in the transcriptomes of mouse brain between acute and chronic *T. gondii* infection (17), and this could alter cytokine responses of the host to infection.

The transcriptional landscape of infected cat tissues was dominated by an immune gene expression signature, wherein, cytokine-cytokine receptor interaction, Jak-STAT signaling pathway, NOD-like receptor signaling pathway, NF-kappa B signaling pathway, MAPK signaling pathway, T cell receptor signaling pathway and the cytosolic DNA sensing pathway, were amongst the up-regulated immune pathways in almost all tissues. The importance of Toll-like receptor signaling in controlling *T. gondii* infection has been established (37). Many of the upregulated genes (e.g., CXCL10, SOCS3, MAPK13, CXCL9, CD2, CSF2RA, PI4K2B, IGF1, PFKFB1, MMP7, FZD8, TNFSF10, and RelB) identified in the livers are involved in immune-related pathways. Some of these genes are involved in the development of Natural Killer (NK) and adaptive T cell responses, leading to the production of Interferon gamma (IFN-γ) and resistance to infection (38). *T. gondii* is very efficient in manipulating the host's immune defense (39, 40); and previous studies have indicated that host immune response is a key determinant of clinical outcome following infection with *T. gondii* (16–20, 41, 42).

Although spleen had the lowest number of DEGs (80 genes) compared to other tissues, KEGG analysis identified multiple immune signaling pathways that were influenced by infection. Most of the immune pathways were up-regulated in spleen of infected cats; such as Toll-like receptor signaling pathway, cytosolic DNA-sensing pathway, RIG-I-like receptor signaling, TNF signaling pathway, FoxO signaling pathway, chemokine signaling pathway, and PI3K/Akt signaling pathway. Of note, C-X-C motif chemokine 10 (CXCL10) was involved in most of the immune-related pathways. Our previous study demonstrated that CXCL10 was up-regulated in pig peripheral blood mononuclear cells during early *T. gondii* infection (20, 21). CXCL10 is a chemokine secreted from cells stimulated with IFN-γ, and plays an important role in chemo-attraction of immune cells (43). This result indicates that *T. gondii* influences chemokine gene expression in spleen during early *T. gondii* infection.

*T. gondii* is a food-borne pathogen and infection is initially established in the small intestine by consuming prey containing parasite cysts or oocyst-contaminated water (44); leading to enteropathy (45). Therefore, successful infection of the host intestine is essential for subsequent parasite dissemination to different tissues. Others have showed that *in vitro* infection of rat intestinal epithelial cells can trigger an inflammatory response characterized by Tumor Necrosis Factor alpha (TNFα) signaling via NF-κB (46). However, feline host factors that are influenced by intestinal infection remain largely unknown. In our study, the global transcriptomic changes in the intestine of the definitive feline host, have been investigated. A total of 130 DEGs in the small intestine of infected cats were identified; of which 75 were down-regulated and 55 up-regulated. Some of the DEGs were involved in immune processes and signaling pathways, including LOC100302541, TNFSF18, CCL20, TNFRSF6B, IFNG, SOCS3, ICAM1, CD36, and FGF19. Other immune pathways up-regulated in liver and spleen tissues were also up-regulated in the small intestine.

An earlier study investigating the transcriptome of mouse brain during *T. gondii* infection reported an increased expression of genes involved in immune responses and cell activation (16). Also, host immune and inflammatory response was the major feature of genes affected by *T. gondii* infection of mouse peritoneal cells at five days post infection (47). We also showed that the expression of genes and signaling pathways involved in host immune response and cell fate, such as PI3K-Akt signaling pathway, Hippo signaling pathway and MAPK signaling pathway, was altered in the cat brain. Transcriptional signatures observed in the cat brain tissue showed also that Notch signaling pathway is involved in *T. gondii* neuro-pathogenesis. Previous *T. gondii* host/pathogen KEGG pathway interactome analysis suggested the involvement of six genes of the Notch pathway in psychiatric/neurological disorders (45, 48). Notch signaling interacts with other signaling pathways, including phosphatidylinositol-3-kinase (PI3K)/serine/threonine kinase (Akt) and NF-κB to regulate cell fate. PI3K/Akt signaling pathway regulates diverse cellular activities related to cell growth, metabolism, migration, and apoptosis (49). Notch signaling plays an important role in various facets of *T. gondii* pathogenesis (50). The possibility that PI3K/Akt signaling pathway participates in promoting *T. gondii* survival and proliferation (51) and in mediating cell survival and blockage of apoptotic responses during *T. gondii* infection (50), suggests a link between *T. gondii* and this signal pathway. This finding supports an earlier observation wherein many *T. gondii* strains were found able to regulate genes enriched for processes involved in cell cycle regulation in murine macrophages (15).

*T. gondii* exploits heterotrimeric Gi-protein-mediated signaling to activate PI3K, leading to phosphorylation of downstream serine/threonine kinase AKT and extracellular signal-regulated protein kinases 1/2 (ERK1/2), and the inhibition of apoptosis (52). In our study, down-regulation of PI3K/AKT signaling in all tissues, except spleen, was detected and this can enhance apoptosis of host tissue and limits parasite growth. Phosphatase (a negative regulator of PI3K/Akt signaling, by converting PIP3 to PIP2, an opposite action to PI3K) can interfere with a number of cellular functions, such as cell proliferation and cell-cycle progression (53), was co-expressed in five cat tissues. These results indicate diverse roles played by PI3K/Akt signaling in *T. gondii*-host interaction. Further investigation into how the PI3K/Akt pathway interacts with other signaling mediators is required. NF-κB and mitogen-activated protein kinase (MAPK) signaling pathways are involved in the innate immune response to *T. gondii* (54). Altered MAPK signaling has been also implicated in toxoplasmosis of mice (55, 56) and humans (57). Considering that NF-κB and MAPK are downstream effectors of Akt, it is of significance to clarify whether this pathway influences the fate of infected cells via the regulation of NF-κB and MAPK.

### Gene co-association across tissues

Co-expression analysis of DEGs indicated that phosphatase gene expression overlapped in five tissues; including brain, heart, liver, small intestine, and spleen. Transcripts for the inner-membrane complex (IMC) protein phosphatase have been involved in gene expression and cell division (46). Indoleamine 2,3-dioxygenase (IDO) was also co-expressed in five tissues: heart, liver, lung, small intestine, and spleen, but not in the brain tissue. IDO, the rate limiting catabolic enzyme in the degradation pathway of tryptophan, initiates the production of tryptophan degradation products, which exert important immuno-regulatory functions. IDO, through T-cell functions and other mechanisms (58), modulates pathophysiological processes, such as antimicrobial and antioxidant activities, and immune-regulation. IFNγ produced in response to *T. gondii* infection induces IDO enzyme to degrade L-tryptophan, an amino acid for which *T. gondii* is auxotrophic (59, 60). IFNγ-induced L-tryptophan starvation was believed to trigger *T. gondii* clearance via noncanonical, ubiquitin-mediated autophagy (61). Guanylate-binding protein (GBP) was detected in the heart, liver, lung, and spleen. GBP, IFN-γ-inducible effector, is a member of the GTPase family, and plays an essential role in mediating host defense against *T. gondii* (62). Human guanylate-binding protein 1 (hGBP1) functions against *T. gondii* infection in human MSCs (hMSCs). *T. gondii* replication can be significantly inhibited by the recruitment of hGBP1 to the parasitophorous vacuole (PV) membrane in IFN-γ-stimulated hMSCs (63). In mice, the recruitment of mGBP2 to *T. gondii*-containing PV was essential for controlling *T. gondii* replication (64).

### Patterns of gene clusters

Genes clustered according to their pattern of expressions across the six tissues examined, led to the identification of seven clusters featuring expressions differentiated across tissues and infected/non-infected conditions. We also detected tissue-specific variations in the percentage of clustered genes, and in the properties of gene clusters including functional annotation and magnitude of gene expression. The differences in each tissue's response to infection may imply that some tissue-specific defense mechanisms exist in order to maintain the balance between enhancing the host's response to infection and promoting the parasite's survival. The genes within these clusters after a further processing step imposing a stringent expression cutoff to avoid any background expression noise were analyzed for differential transcriptional changes between infected and uninfected samples (magnitude). Interestingly, the lung besides showing the greater number of genes showed also the largest magnitude of transcriptional changes between infected and non-infected conditions, suggesting that changes induced by infection in lungs seem to rely on genes, which had large transcriptional changes.

## Conclusion

We used two complementary approaches to characterize alterations in tissue-specific gene expression in cats infected with *T. gondii*. Our results revealed considerable transcriptional differences between cat body tissues, and between infected and healthy cats. We identified significant tissue-specific differences in gene expression, and in gene cluster content and functional annotations. The differences in gene expression and gene clusters may result from tissue-specific differences in the defense processes that shape the host-pathogen interaction. Our data also underlined the importance of immune and inflammatory response to *T. gondii* infection, regardless of tissue types. Genes and pathways discovered in this study, should serve as a basis for further understanding the cellular and molecular basis of cat response to *T. gondii*. These results may assist the selection of biomarkers useful for developing new diagnostic tools or therapeutic interventions to control toxoplasmosis in cats.

## Accession number(s)

The RNA-seq data reported in this study have been deposited in GenBank's Short Read Archive (SRA) database under BioProject number PRJNA296557.

## Author contributions

X-QZ, HE, and WC designed the experiment. WC supervised the experimental infection. WC, F-KZ, C-XZ, J-JH, and X-XZ performed the experiments. WC, HE, TD, FK, and RE contributed reagents, materials, and analysis tools. TD, FK, and RE developed computational algorithms and performed the bioinformatics analysis. WC, HE, and X-QZ wrote the paper. All authors commented on the manuscript.

### Conflict of interest statement

The authors declare that the research was conducted in the absence of any commercial or financial relationships that could be construed as a potential conflict of interest.

## Supplementary material

Figure S1 Agarose gel electrophoresis of PCR amplicons after nested amplification of *Toxoplasma gondii B1* gene-specific fragment from cat tissue DNA. ~96-bp products of *B1* gene were amplified from ~193-bp *B1* PCR products, originally generated from genomic DNA extracted from different cat tissues, using the nested primers 5′-TGCATAGGTTGCAGTCACTG-3′ and 5′-GGCGACCAATCTGCGAATACACC-3′. Samples were analyzed by electrophoresis through 2% (wt/vol) agarose gels. Gels were stained with ethidium bromide and DNA was visualized under UV. Lanes: M, DNA ladder marker (TAKARA, China); 1, positive control; 2, negative control without DNA template; 3–8, positive PCR products from brain, heart, liver, lung, spleen and small intestine of infected cats; 9–14, negative results of samples obtained from the equivalent tissues of uninfected cats. The numbers to the left refer to the size (bp) of marker DNA fragments.

Figure S2 Differential gene expression patterns across tissues and GO terms of the overlapping DEGs. **(A)** A heatmap of the genes expressed in all six tissues. **(B)** Gene Ontology terms associated with the co-expressed DEGs.

Figure S3 Gene Ontology terms and KEGG pathways distribution per gene cluster.

Table S1 Summary of RNA-Sequencing data obtained by Illumina HiSeq 2500 platform.

Table S2 Summary of read mapping.

Table S3 The number of gene in different expression level intervals.

Table S4 Differentially expressed genes in various cat tissues.

Table S5 Functional enrichment analysis of DEGs in various cat tissues.

Table S6 DEGs involved in immune-related pathways in cat tissues.

Table S7 Differentially co-expressed genes across various cat tissues.

Table S8 Gene Ontology analysis of seven gene clusters.
