# Supplementary figures and images for: Acute Toxoplasma Gondii Infection in Cats Induced Tissue-Specific Transcriptional Response Dominated by Immune Signatures
Source: Front Immunol. 2018 Oct 19;9:2403. doi: 10.3389/fimmu.2018.02403 (PMC6202952; doi:10.3389/fimmu.2018.02403)

**M**

1

2

3

4

5

6

7

8

9

10

11

12

13

14

2 kb

1 kb

750 bp

500 bp

250 bp

100 bp

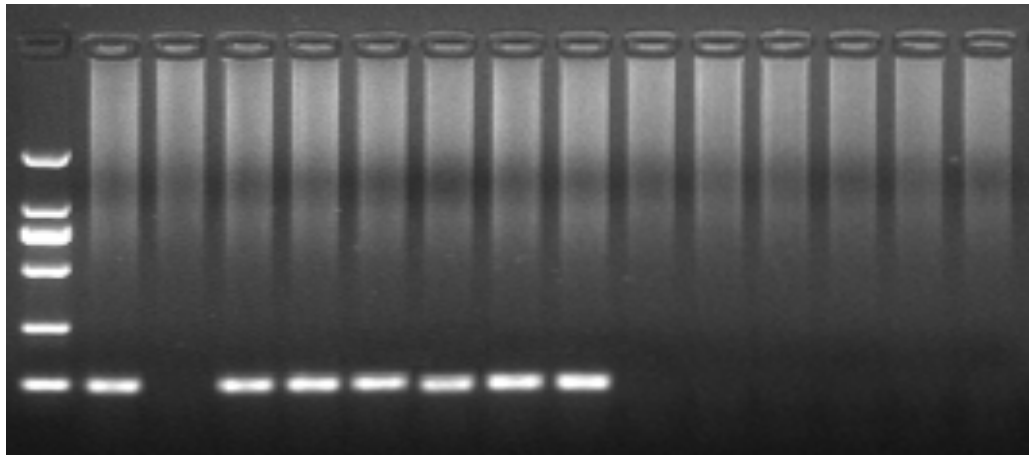

Supplement: Figure S1 — Agarose gel electrophoresis of PCR amplicons after nested amplification of Toxoplasma gondii B1 gene-specific fragment from cat tissue DNA. ~96-bp products of B1 gene were amplified from ~193-bp B1 PCR products, originally generated from genomic DNA extracted from different cat tissues, using the nested primers 5′-TGCATAGGTTGCAGTCACTG-3′ and 5′-GGCGACCAATCTGCGAATACACC-3′. Samples were analyzed by electrophoresis through 2% (wt/vol) agarose gels. Gels were stained with ethidium bromide and DNA was visualized under UV. Lanes: M, DNA ladder marker (TAKARA, China); 1, positive control; 2, negative control without DNA template; 3–8, positive PCR products from brain, heart, liver, lung, spleen and small intestine of infected cats; 9–14, negative results of samples obtained from the equivalent tissues of uninfected cats. The numbers to the left refer to the size (bp) of marker DNA fragments. [file Data_Sheet_1.PDF]

A

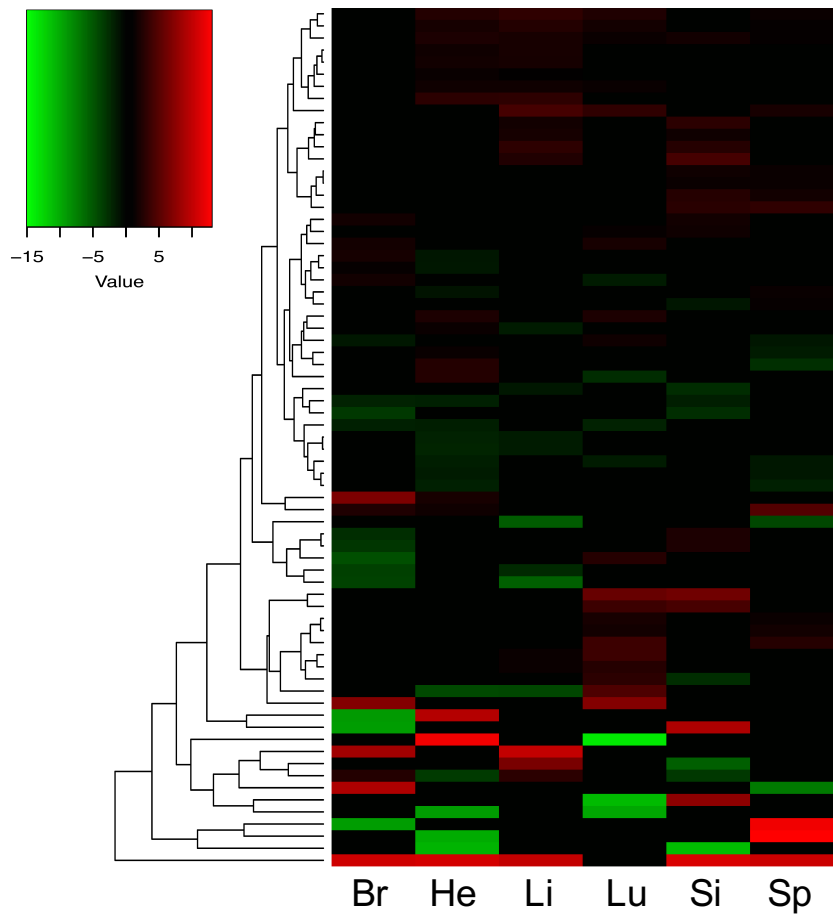

B

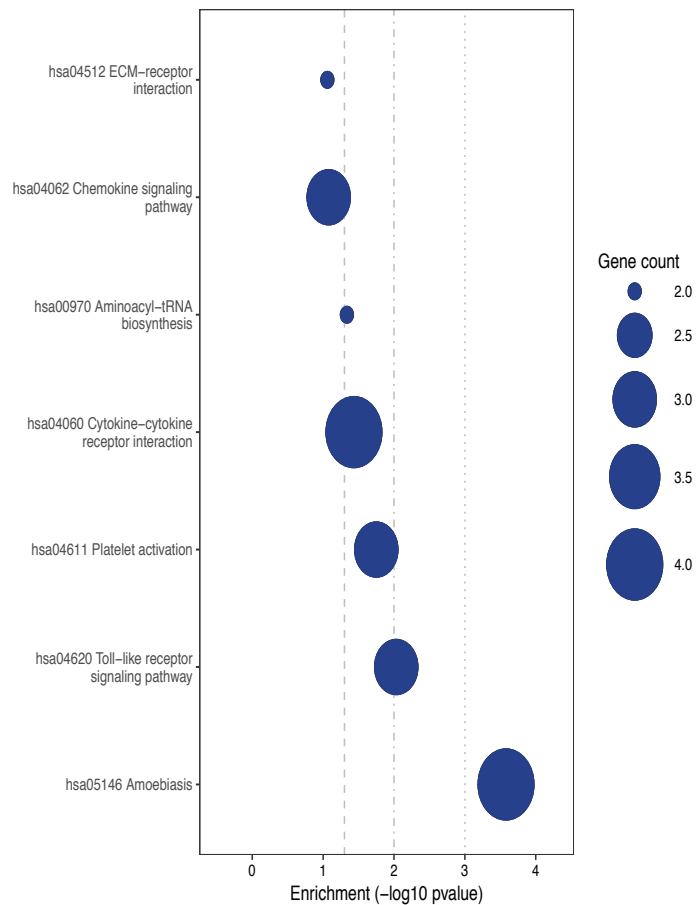

Supplement: Figure S2 — Differential gene expression patterns across tissues and GO terms of the overlapping DEGs. (A) A heatmap of the genes expressed in all six tissues. (B) Gene Ontology terms associated with the co-expressed DEGs. [file Data_Sheet_2.PDF]

GO terms and KEGG pathways

BP CC MF KEGG

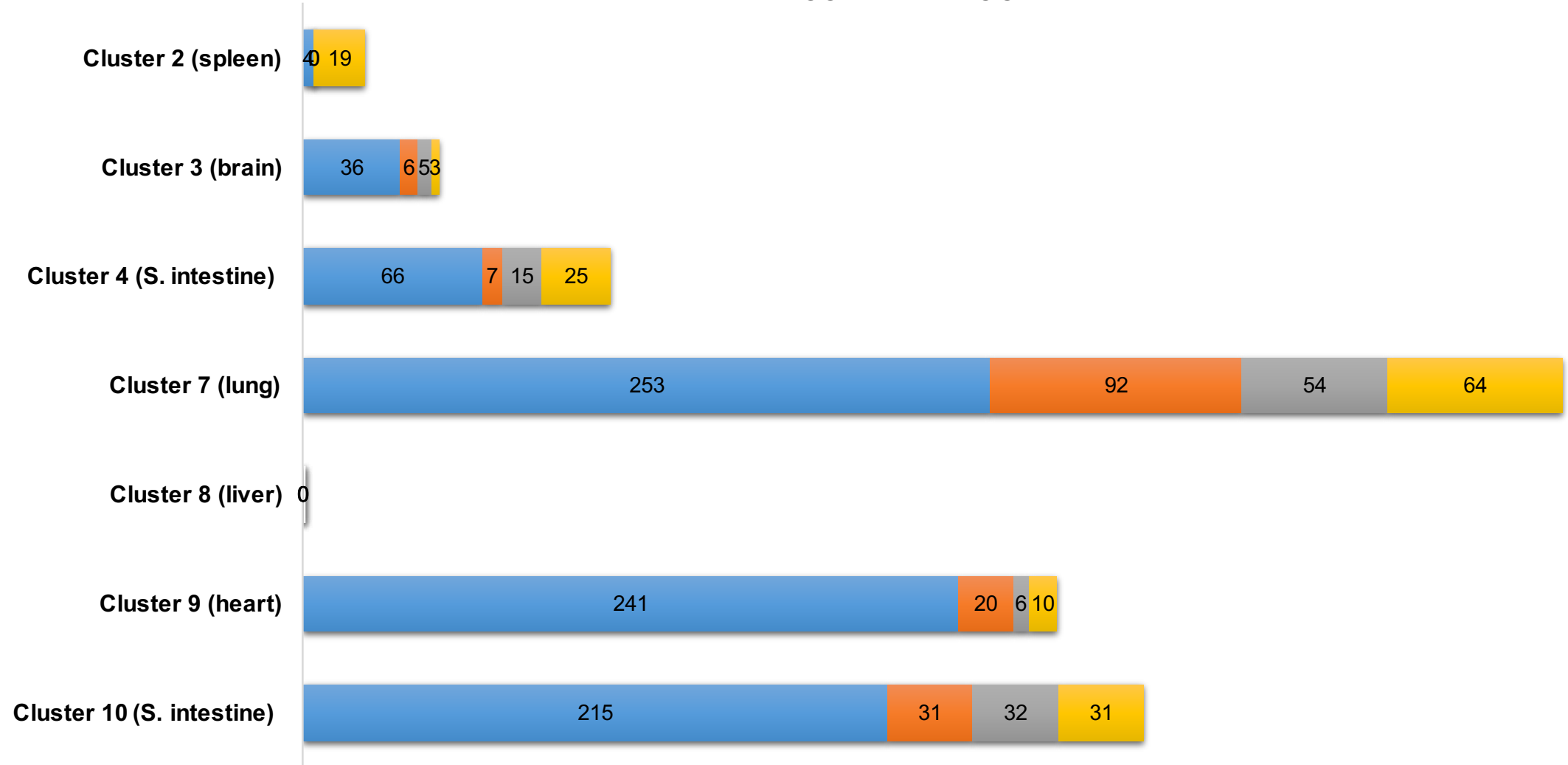

Supplement: Figure S3 — Gene Ontology terms and KEGG pathways distribution per gene cluster. [file Data_Sheet_3.PDF]
