# Supplementary material for: Acute Toxoplasma Gondii Infection in Cats Induced Tissue-Specific Transcriptional Response Dominated by Immune Signatures
Source: Front Immunol. 2018 Oct 19;9:2403. doi: 10.3389/fimmu.2018.02403 (PMC6202952; doi:10.3389/fimmu.2018.02403)
Supplement: Table S1 — Summary of RNA-Sequencing data obtained by Illumina HiSeq 2500 platform. [file Table_1.DOCX]

**Table S1** Summary of RNA-Sequencing data obtained by Illumina HiSeq 2500 platform.

| Sample  ID | Raw  reads | Clean  reads | Clean bases | Error rate (%) | Q20  (%) | Q30 (%) | GC content (%) |
| --- | --- | --- | --- | --- | --- | --- | --- |
| **Brain** |  |  |  |  |  |  |  |
| Uninfected 1 | 57826825 | 55104494 | 6.88 G | 0.035 | 95.22 | 90.96 | 52.10 |
| Uninfected 2 | 50426284 | 48611760 | 6.08 G | 0.035 | 95.11 | 90.77 | 50.30 |
| Infected 1 | 59594436 | 56865752 | 7.10 G | 0.035 | 95.40 | 91.23 | 52.49 |
| Infected 2 | 54051594 | 51088346 | 6.38 G | 0.035 | 95.34 | 91.12 | 52.74 |
| **Heart** |  |  |  |  |  |  |  |
| Uninfected 1 | 59604616 | 57018386 | 7.12 G | 0.035 | 95.34 | 91.10 | 50.85 |
| Uninfected 2 | 57277914 | 54321564 | 6.80 G | 0.035 | 95.19 | 90.81 | 51.07 |
| Infected 1 | 50326646 | 48817578 | 6.10 G | 0.035 | 95.40 | 91.20 | 50.18 |
| Infected 2 | 51493982 | 49923546 | 6.24 G | 0.035 | 95.37 | 91.18 | 49.67 |
| **Liver** |  |  |  |  |  |  |  |
| Uninfected 1 | 60955142 | 58893852 | 7.36 G | 0.035 | 95.44 | 91.21 | 51.80 |
| Uninfected 2 | 62907346 | 57824114 | 7.22 G | 0.035 | 95.45 | 91.27 | 52.09 |
| Infected 1 | 65389556 | 59317326 | 7.42 G | 0.035 | 95.39 | 91.19 | 52.19 |
| Infected 2 | 65273832 | 59030634 | 7.38 G | 0.035 | 95.44 | 91.28 | 52.44 |
| **Lung** |  |  |  |  |  |  |  |
| Uninfected 1 | 62614848 | 57117488 | 7.14 G | 0.035 | 95.14 | 90.81 | 52.36 |
| Uninfected 2 | 57967252 | 54962918 | 6.88 G | 0.035 | 95.19 | 90.90 | 51.65 |
| Infected 1 | 59254748 | 54186620 | 6.78 G | 0.035 | 95.15 | 90.82 | 52.31 |
| Infected 2 | 50440892 | 48073388 | 6.00 G | 0.035 | 95.08 | 90.81 | 51.28 |
| **Small intestine** |  |  |  |  |  |  |  |
| Uninfected 1 | 60764330 | 57731700 | 7.22 G | 0.035 | 95.15 | 90.79 | 51.71 |
| Uninfected 2 | 79438050 | 75611292 | 9.46 G | 0.035 | 94.94 | 90.43 | 51.33 |
| Infected 1 | 71209636 | 67266364 | 8.48 G | 0.035 | 95.21 | 90.89 | 51.60 |
| Infected 2 | 72205928 | 70249018 | 8.78 G | 0.035 | 95.19 | 90.83 | 51.67 |
| **Spleen** |  |  |  |  |  |  |  |
| Uninfected 1 | 61168440 | 58192964 | 7.28 G | 0.035 | 95.40 | 91.23 | 52.38 |
| Uninfected 2 | 56202510 | 52987160 | 6.62 G | 0.035 | 95.22 | 90.96 | 52.18 |
| Infected 1 | 53805588 | 50895990 | 6.36 G | 0.035 | 95.35 | 91.15 | 52.81 |
| Infected 2 | 53499370 | 51522238 | 6.44 G | 0.035 | 95.41 | 91.28 | 50.62 |

Q20, percentage of bases with a Phred value >20; Q30, percentage of bases with a Phred value >30
