# Supplementary material for: Acute Toxoplasma Gondii Infection in Cats Induced Tissue-Specific Transcriptional Response Dominated by Immune Signatures
Source: Front Immunol. 2018 Oct 19;9:2403. doi: 10.3389/fimmu.2018.02403 (PMC6202952; doi:10.3389/fimmu.2018.02403)
Supplement: Table S2 — Summary of read mapping. [file Table_2.DOCX]

**Table S2.** Summary of reads mapping.

| Sample ID | Total  reads | Total  mapped | Multiple  mapped | Uniquely mapped | Reads map to '+' | Reads map to '-' | Non-splice reads | Splice  reads |
| --- | --- | --- | --- | --- | --- | --- | --- | --- |
| **Brain** |  |  |  |  |  |  |  |  |
| UnBr 1 | 55104494 | 45550115  (82.66%) | 349617  (0.63%) | 45200498 (82.03%) | 22604998 (41.02%) | 22595500 (41.00%) | 30363205 (55.1%) | 14837293 (26.93%) |
| UnBr 2 | 48611760 | 40268328 (82.84%) | 296296 (0.61%) | 39972032 (82.23%) | 19980862 (41.10%) | 19991170 (41.12%) | 27342738 (56.25%) | 12629294 (25.98%) |
| InBr 1 | 56865752 | 46537258 (81.84%) | 364201 (0.64%) | 46173057 (81.20%) | 23083665 (40.59%) | 23089392 (40.60%) | 30999143 (54.51%) | 15173914 (26.68%) |
| InBr 2 | 51088346 | 41788298 (81.80%) | 332281 (0.65%) | 41456017 (81.15%) | 20729587 (40.58%) | 20726430 (40.57%) | 27461703 (53.75%) | 13994314 (27.39%) |
| **Heart** |  |  |  |  |  |  |  |  |
| UnHe 1 | 57018386 | 47732024 (83.71%) | 569724 (1%) | 47162300 (82.71%) | 23617974 (41.42%) | 23544326 (41.29%) | 29245783 (51.29%) | 17916517 (31.42%) |
| UnHe 2 | 54321564 | 45220955 (83.25%) | 501101 (0.92%) | 44719854 (82.32%) | 22392212 (41.22%) | 22327642 (41.10%) | 27620097 (50.85%) | 17099757 (31.48%) |
| InHe 1 | 48817578 | 40813987 (83.61%) | 418693 (0.86%) | 40395294 (82.75%) | 20218764 (41.42%) | 20176530 (41.33%) | 25431854 (52.10%) | 14963440 (30.65%) |
| InHe 2 | 49923546 | 42032764 (84.19%) | 431976 (0.87%) | 41600788 (83.33%) | 20830835 (41.73%) | 20769953 (41.60%) | 25991241 (52.06%) | 15609547 (31.27%) |
| **Liver** |  |  |  |  |  |  |  |  |
| UnLi 1 | 58893852 | 49399317 (83.88%) | 630086 (1.07%) | 48769231 (82.81%) | 24468489 (41.55%) | 24300742 (41.26%) | 23815414 (40.44%) | 24953817 (42.37%) |
| UnLi 2 | 57824114 | 48573947 (84.00%) | 737851 (1.28%) | 47836096 (82.73%) | 23964436 (41.44%) | 23871660 (41.28%) | 23672754 (40.94%) | 24163342 (41.79%) |
| InLi 1 | 59317326 | 49304418 (83.12%) | 746598 (1.26%) | 48557820 (81.86%) | 24326419 (41.01%) | 24231401 (40.85%) | 24537655 (41.37%) | 24020165 (40.49%) |
| InLi 2 | 59030634 | 49381732 (83.65%) | 662059 (1.12%) | 48719673 (82.53%) | 24408102 (41.35%) | 24311571 (41.18%) | 24655236 (41.77%) | 24064437 (40.77%) |
| **Lung** |  |  |  |  |  |  |  |  |
| UnLu 1 | 57117488 | 47440014 (83.06%) | 451406 (0.79%) | 46988608 (82.27%) | 23504677 (41.15%) | 23483931 (41.12%) | 29981230 (52.49%) | 17007378 (29.78%) |
| UnLu 2 | 54962918 | 45794309 (83.32%) | 416057 (0.76%) | 45378252 (82.56%) | 22697950 (41.30%) | 22680302 (41.26%) | 28978287 (52.72%) | 16399965 (29.84%) |
| InLu 1 | 54186620 | 45083479 (83.20%) | 429762 (0.79%) | 44653717 (82.41%) | 22332048 (41.21%) | 22321669 (41.19%) | 28375997 (52.37%) | 16277720 (30.04%) |
| InLu 2 | 48073388 | 39947424 (83.10%) | 384454 (0.8%) | 39562970 (82.30%) | 19789718 (41.17%) | 19773252 (41.13%) | 26098425 (54.29%) | 13464545 (28.01%) |
| **Small intestine** |  |  |  |  |  |  |  |  |
| UnSi 1 | 57731700 | 47794592 (82.79%) | 506421 (0.88%) | 47288171 (81.91%) | 23660692 (40.98%) | 23627479 (40.93%) | 28260836 (48.95%) | 19027335 (32.96%) |
| UnSi 2 | 75611292 | 62654408 (82.86%) | 701234 (0.93%) | 61953174 (81.94%) | 30992800 (40.99%) | 30960374 (40.95%) | 36216253 (47.90%) | 25736921 (34.04%) |
| InSi 1 | 67266364 | 54929949 (80.94%) | 650171 (0.96%) | 54279778 (79.98%) | 27160561 (40.02%) | 27119217 (39.96%) | 31561484 (46.51%) | 22718294 (33.48%) |
| InSi 2 | 70249018 | 56260350 (80.09%) | 633756 (0.9%) | 55626594 (79.18%) | 27841583 (39.63%) | 27785011 (39.55%) | 31753117 (45.20%) | 23873477 (33.98%) |
| **Spleen** |  |  |  |  |  |  |  |  |
| UnSp 1 | 58192964 | 48242735 (82.90%) | 607811 (1.04%) | 47634924 (81.86%) | 23830329 (40.95%) | 23804595 (40.91%) | 29552512 (50.78%) | 18082412 (31.07%) |
| UnSp2 | 52987160 | 43784563 (82.63%) | 490440 (0.93%) | 43294123 (81.71%) | 21645532 (40.85%) | 21648591 (40.86%) | 27518437 (51.93%) | 15775686 (29.77%) |
| InSp 1 | 50895990 | 41713452 (81.96%) | 532739 (1.05%) | 41180713 (80.91%) | 20588434 (40.45%) | 20592279 (40.46%) | 25574019 (50.25%) | 15606694 (30.66%) |
| InSp 2 | 51522238 | 43005709 (83.47%) | 495428 (0.96%) | 42510281 (82.51%) | 21252161 (41.25%) | 21258120 (41.26%) | 27229184 (52.85%) | 15281097 (29.66%) |
