# Supplementary material for: Acute Toxoplasma Gondii Infection in Cats Induced Tissue-Specific Transcriptional Response Dominated by Immune Signatures
Source: Front Immunol. 2018 Oct 19;9:2403. doi: 10.3389/fimmu.2018.02403 (PMC6202952; doi:10.3389/fimmu.2018.02403)
Supplement: Table S3 — The number of gene in different expression level intervals. [file Table_3.DOCX]

**Table S3. The number of genes by different expression level intervals.**

| **FPKM Interval** | **0~1** | **1~3** | **3~15** | **15~60** | **>60** |
| --- | --- | --- | --- | --- | --- |
| **Brain** |  |  |  |  |  |
| UnBr 1 | 12066 (43.34%) | 3062 (11.00%) | 6449 (23.16%) | 4508 (16.19%) | 1758 (6.31%) |
| UnBr 2 | 12130 (43.57%) | 3060 (10.99%) | 6408 (23.01%) | 4561 (16.38%) | 1684 (6.05%) |
| InBr 1 | 11846 (42.55%) | 3163 (11.36%) | 6550 (23.52%) | 4510 (16.20%) | 1774 (6.37%) |
| InBr 2 | 12080 (43.39%) | 3057 (10.98%) | 6330 (22.73%) | 4564 (16.39%) | 1812 (6.51%) |
| **Heart** |  |  |  |  |  |
| UnHe 1 | 14308 (51.39%) | 3161 (11.35%) | 6220 (22.34%) | 2985 (10.72%) | 1169 (4.20%) |
| UnHe 2 | 14259 (51.21%) | 3163 (11.36%) | 6173 (22.17%) | 3066 (11.01%) | 1182 (4.25%) |
| InHe 1 | 14121 (50.72%) | 3085 (11.08%) | 6287 (22.58%) | 3149 (11.31%) | 1201 (4.31%) |
| InHe 2 | 13953 (50.11%) | 3069 (11.02%) | 6337 (22.76%) | 3261 (11.71%) | 1223 (4.39%) |
| **Liver** |  |  |  |  |  |
| UnLi 1 | 15480 (55.60%) | 3298 (11.84%) | 5577 (20.03%) | 2366 (8.50%) | 1122 (4.03%) |
| UnLi 2 | 15648 (56.20%) | 3180 (11.42%) | 5486 (19.70%) | 2401 (8.62%) | 1128 (4.05%) |
| InLi 1 | 15507 (55.69%) | 3166 (11.37%) | 5493 (19.73%) | 2458 (8.83%) | 1219 (4.38%) |
| InLi 2 | 15301 (54.95%) | 3056 (10.98%) | 5659 (20.32%) | 2591 (9.31%) | 1236 (4.44%) |
| **Lung** |  |  |  |  |  |
| UnLu 1 | 11904 (42.75%) | 3050 (10.95%) | 6289 (22.59%) | 4874 (17.51%) | 1726 (6.20%) |
| UnLu 2 | 12051 (43.28%) | 2979 (10.70%) | 6238 (22.40%) | 4853 (17.43%) | 1722 (6.18%) |
| InLu 1 | 11834 (42.50%) | 3045 (10.94%) | 6298 (22.62%) | 4917 (17.66%) | 1749 (6.28%) |
| InLu 2 | 11109 (39.90%) | 3088 (11.09%) | 6824 (24.51%) | 5037 (18.09%) | 1785 (6.41%) |
| **Small intestine** |  |  |  |  |  |
| UnSi 1 | 12862 (46.19%) | 2983 (10.71%) | 6191 (22.24%) | 4026 (14.46%) | 1781 (6.40%) |
| UnSi 2 | 12979 (46.61%) | 2971 (10.67%) | 6015 (21.60%) | 4069 (14.61%) | 1809 (6.50%) |
| InSi 1 | 12808 (46.00%) | 2848 (10.23%) | 6136 (22.04%) | 4205 (15.10%) | 1846 (6.63%) |
| InSi 2 | 13484 (48.43%) | 2835 (10.18%) | 5824 (20.92%) | 3889 (13.97%) | 1811 (6.50%) |
| **Spleen** |  |  |  |  |  |
| UnSp 1 | 12655 (45.45%) | 2901 (10.42%) | 6129 (22.01%) | 4417 (15.86%) | 1741 (6.25%) |
| UnSp2 | 12223 (43.90%) | 3002 (10.78%) | 6046 (21.71%) | 4796 (17.23%) | 1776 (6.38%) |
| InSp 1 | 12461 (44.75%) | 3055 (10.97%) | 6004 (21.56%) | 4563 (16.39%) | 1760 (6.32%) |
| InSp 2 | 12358 (44.38%) | 2927 (10.51%) | 5949 (21.37%) | 4879 (17.52%) | 1730 (6.21%) |
